# *De Novo* Biosynthesis of Apigenin, Luteolin, and Eriodictyol in the Actinomycete *Streptomyces albus* and Production Improvement by Feeding and Spore Conditioning

**DOI:** 10.3389/fmicb.2017.00921

**Published:** 2017-05-30

**Authors:** Laura Marín, Ignacio Gutiérrez-del-Río, Paula Yagüe, Ángel Manteca, Claudio J. Villar, Felipe Lombó

**Affiliations:** Biotechnology in Nutraceuticals and Bioactive Compounds-BIONUC, Departamento de Biología Funcional, Área de Microbiología, University of OviedoOviedo, Spain

**Keywords:** flavonoid, flavone, flavanone, polyphenol, nutraceutical, antioxidant, anti-inflammatory

## Abstract

Nutraceutical compounds as plant flavonoids play an important role in prevention and modulation of diverse heath conditions, as they exert interesting antifungal, antibacterial, antioxidant, and antitumor effects. They also possess anti-inflammatory activities in arthritis, cardiovascular disease or neurological diseases, as well as modulatory effects on the CYP450 activity on diverse drugs. Most flavonoids are bioactive molecules of plant origin, but their industrial production is sometimes hindered due to reasons as low concentration in the plant tissues, presence in only some species or as a complex mixture or inactive glycosides in plant vacuolae. In this work, we describe the *de novo* biosynthesis of two important flavones, apigenin and luteolin, and one known flavanone, eriodictyol. Their plant biosynthetic pathways have been reconstructed for heterologous expression in *Streptomyces albus*, an actinomycete bacterium manageable at industrial production level. Also, production levels for apigenin have been improved by feeding with naringenin precursor, and timing for settlement of secondary metabolism has been advanced by spore conditioning. In the cases of eriodictyol and luteolin, their production in this important type of biotechnology-prone bacteria, the actinomycetes, had not been described in the literature yet.

## Introduction

Polyphenols are one of the largest and widely natural products distributed in plant cells, since more than 10,000 phenolic compounds have been described so far in higher plants, with several 100 found in edible plants (Manach et al., 2004; Tsao, 2010; Li et al., 2014). Flavonoids (Latin flavus, “yellow”) represent 60% of these polyphenols and cover more than 6000 compounds ubiquitously distributed in plants (Chaudhuri et al., 2013; González-Vallinas et al., 2013).

**FIGURE 1:**
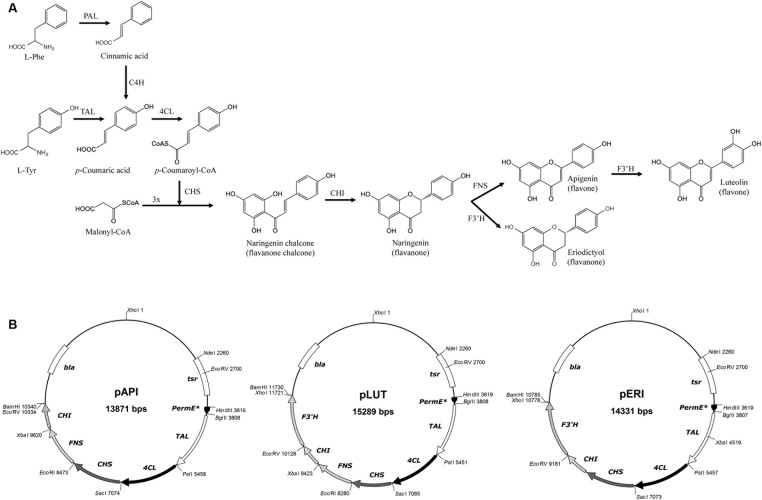
**(A)** Biosynthetic pathway for biosynthesis of the flavones apigenin and luteolin and the flavanone eriodictyol in plants (using PAL and C4H) and in *Streptomyces albus* (suing TAL). **(B)** Recombinant plasmids constructed in this work for the heterologous biosynthesis of these flavonoids.

All flavonoids have a generic chemical structure consisting of 15 carbon atoms (C6-C3-C6): two aromatic rings (rings A and B) connected by a heterocyclic pyran C which contains one oxygen (ring C, **Figure 1**) (Kumar and Pandey, 2007; Verma and Pratap, 2010; Liu, 2013; Ravishankar et al., 2013; Fantini et al., 2015). This basic skeleton can have multiple substituents, as hydroxyl groups as well as sugars (Crozier et al., 2009). Depending on the pattern of hydroxylation and the substituents on the heterocyclic ring C, flavonoids can be classified into several sub-groups, but in this paper we focus on two groups: flavones as luteolin (present in Thai chili, broccoli and leaves of onion and celery) and apigenin (present in parsley, celery, onion, garlic, red pepper, and chamomile tea); and flavanones (as eriodictyol found in citrus fruits such as orange, lemon, or grapefruit) (Manach et al., 2004; Tsao, 2010; Liu, 2013).

The biological activities of these compounds depend both on their structural differences and glycosylation patterns (Kumar and Pandey, 2007; Tsao, 2010). In fact, diet flavonoids can promote health and prevent certain diseases such as cancer by acting against cell oxidation processes and by stopping cell degradation and aging (Luo et al., 2015).

The effects of flavonoids in humans can be attributed to antioxidant, antitumor, anti-inflammatory, antimicrobial, anticancer, and cardiovascular actions. For example, eriodictyol performs a key role against the pathogenesis of diabetes mellitus, inhibiting immunoglobulin E/antigen-produced type I hypersensitivity. This flavanone also shows antinociceptive, hyperthermic, and anticancer effects (Zhu L. et al., 2014). Apigenin has a growing importance due to its inhibitory effect on the PI3K/Akt/mTOR signaling pathways (Tong and Pelling, 2013). Luteolin is able to inhibit angiogenesis or to reduce tumor size *in vivo* since it is able to modulate protein kinases and growth factor receptors as EGFRs or VEGFRs (López-Lázaro, 2006). Also, the antioxidant capacity of these and other polyphenols acts as master key in preventing numerous diseases associated with oxidative damage such as cardiovascular, neurodegenerative and cancer (Ravishankar et al., 2013; Hollman, 2014).

Flavonoids are synthesized *in planta* by the phenylpropanoid pathway, which converts L-Phe in 4-coumaroyl-CoA in three steps, the common intermediate for all flavonoids (**Figure 1**) (Falcone Ferreyra et al., 2012). These first three common steps are catalyzed by phenylalanine ammonia lyase (PAL), cinnamate 4-hydroxylase (4CH) and 4-coumaroyl CoA ligase (4CL) (**Figure 1**). Then the chalcone synthase (CHS) condenses a molecule of 4-coumaroyl-CoA with three molecules of malonyl-CoA, generating naringenin chalcone, the basic skeleton for all flavonoids (Tsao, 2010; Wang et al., 2011; Falcone Ferreyra et al., 2012). The heterocycle C closure is catalyzed by chalcone isomerase (CHI), which generates naringenin, the precursor for apigenin and luteolin (flavones) and for eriodictyol (flavanone). In order to generate apigenin, the flavone synthase (FNS) oxidoreductase is required. Apigenin is then the substrate for the flavonoid 3′-hydroxylase (F3′H), giving rise to luteolin (**Figure 1**). On the other hand, naringenin is also the substrate for F3′H, generating eriodictyol without FNS activity (**Figure 1**) (Crozier et al., 2009).

Flavonoids are valuable compounds for use in the pharmaceutical, chemical and nutraceutical industry, but the amounts that can be obtained directly from plants are very limited. One solution is the chemical synthesis, but when dealing with metabolites with complex chemical structures, their production is economically not feasible in most cases. Therefore, an attractive alternative is to express these plant biosynthetic gene pathways in microbial factories, using combinatorial biosynthesis, where genes from different organisms are grouped in an artificial gene cluster directed to the production of the natural bioactive compound (Falcone Ferreyra et al., 2012; Trantas et al., 2015). Microorganisms that are most commonly used as cell factories for flavonoids are *Saccharomyces cerevisiae, Escherichia coli* and in some cases *Streptomyces venezuelae*, where different techniques are applied in order to increase production levels.

However, in the last years, focus has been made in *de novo* processes for flavonoids microbial production, without adding expensive precursors to the culture medium, therefore allowing cheap production platforms necessary for broad commercial use of flavonoids as nutraceuticals (Fowler and Koffas, 2009; Wang et al., 2011; Wu et al., 2014). In this work, we have achieved *de novo* production of apigenin, luteolin, and eriodictyol by means of synthetic biosynthetic pathways heterologous expressed in *Streptomyces albus.* The use of Gram-positive bacterial factories, as this actinomycete, facilitates the further industrialization of bioactive compounds for the pharmaceutical industry, as on the one hand, actinomycetes are the most diverse and rich producers of bioactive compounds (antibacterials, antifungals, antitumors, herbicides, immunosuppressors, etc.), belonging to many different biochemical families (lactams, polyketides, non-ribosomal peptides, terpenoids, flavonoids, etc.), and providing all the necessary metabolic precursors and biosynthetic machinery for these purposes, including also all post-translationally modification enzymes (Newman and Cragg, 2007). Actually, about two thirds of all known bioactive polyketides are produced by actinomycetes. This huge biosynthetic capability is accompanied also by a huge diversity in resistance mechanisms to allow production of such a wide range of bioactive compounds (Zhang et al., 2008; Hwang et al., 2014). This role as useful bacterial factories for bioactives is reinforced in the case of some actinomycetes as *Saccharopolyspora erythraea, S. coelicolor, S. lividans, S. avermitilis, S. venezuelae* and *S. albus*, as their genomes have been sequenced and multiple genetic manipulation tools are available for them. Specifically, in the case of *S. albus*, this host is more convenient as it shows a fast and nice disperse mycelial growth, instead of the most common mycelial dense aggregates shown in the case of other actinomycetes. This disperse growth facilitates scaling-up at industrial bioreactors, as less complications arise from local cellular density and cell lysis. Also, *S. albus* can be transformed with methylated plasmid DNA, in contrast to other actinomycetes, which also facilitates genetic engineering in this species (Kieser et al., 2000).

## Materials and Methods

### Bacterial Strains, Plasmids, and Culture Conditions

*Escherichia coli* TOP10 (Invitrogen) and pUC57 (Fermentas) were used for routine sub-cloning. The high-copy number shuttle vector pIAGO (**Table 1**) for *E. coli*–*Streptomyces*, which contains the strong constitutive *ermE* promoter P_ermE^∗^_ (Aguirrezabalaga et al., 2000), was used as expression plasmid in the strain *S. albus* J1074 (Chater and Wilde, 1980), which was used as host for the production of flavones and flavanones (**Table 2**).

**Table 1:** List of plasmids used in this study.

Plasmid	Description	Source
pIAGO	pWHM3 (replicative shuttle vector) harboring permE^∗^	Aguirrezabalaga et al., 2000
pSL1180	*E. coli* vector	Brosius, 1989
pUC57	*E. coli* vector	Fermentas
pLMF1	pUC57 harboring TAL	This study
pLMF2	pUC57 harboring 4CL	This study
pLMF3	pUC57 harboring CHS	This study
pLMF5	pUC57 harboring CHI	This study
pLMF-FNS	pUC57 harboring FNS	This study
pLMF7	pSL1180 harboring TAL	This study
pLMF8	pSL1180 harboring TAL and 4CL	This study
pNGM1	pSL1180 harboring CHS and FNS	This study
pNGM2	pSL1180 harboring TAL, 4CL, CHS, and FNS	This study
pNGM3	pSL1180 harboring TAL, 4CL, CHS, FNS, and CHI	This study
pAPI	pIAGO harboring TAL, 4CL, CHS, FNS, and CHI	This study
pNGM4	pSL1180 harboring TAL, 4CL, CHS, F3′H, and CHI	This study
pERI	pIAGO harboring TAL, 4CL, CHS, F3′H, and CHI	This study
pNGM5	pSL1180 harboring TAL, 4CL, CHS, FNS, CHI, and F3′H	This study
pLUT	pIAGO harboring TAL, 4CL, CHS, FNS, CHI, and F3′H	This study


**Table 2:** List of strains used in this study.

Strains	Description	Source
*E. coli* TOP10	Strain used for routine sub-cloning and transformation in *S. albus*	Invitrogen
*Streptomyces albus* J1074	Strain used to create the flavonoid-producing mutants	Chater and Wilde, 1980
*S. albus*-pIAGO	*S. albus* harboring pIAGO used as negative control	This study
*S. albus*-pAPI	*S. albus* harboring pAPI	This study
*S. albus*-pERI	*S. albus* harboring pERI	This study
*S. albus*-pLUT	*S. albus* harboring pLUT	This study


*Escherichia coli* was grown at 37°C in TSB liquid broth or TSB agar (VWR), supplemented with the corresponding antibiotics for plasmid selection (ampicillin 100 μg/ml, Sigma Aldrich). *S. albus* J1074 was grown at 30°C in YEME 17% sucrose (Kieser et al., 2000) for the preparation of protoplasts. It was sporulated on Bennet medium (Kieser et al., 2000), and supplemented with the corresponding antibiotics when necessary (thiostrepton 50 μg/ml, Sigma Aldrich).

For flavonoids production, *S. albus* clones were grown in 25 ml of R5A medium (Fernández et al., 1998), supplemented with the corresponding antibiotic, during 184 h (96 h for feeding experiments) at 30°C and at 250 rpm. Spores were previously quantified and an inoculum of 10^7^ spores/ml was used always as inoculum.

### DNA Manipulation

Restriction enzymes were purchased from Takara Biochemicals, T4 DNA ligase, Klenow fragment and Dream Taq DNA Polymerase from Thermo Scientific. Synthetic genes for the following ORFs (EBI preliminary accession number Hx2000056376) were generated by Genecust after codon optimization: TAL (as *Bgl*II-*Pst*I gene cassette) from *Rhodobacter capsulatus* (GenBank accession no. WP_013066811), 4CL (as *Pst*I-*Sac*I gene cassette) from *S. coelicolor* (GenBank accession no. NP_628552), CHS (as *Sac*I-*Eco*RI gene cassette) from *Glycine max* (GenBank accession no. L07647.1), CHI (as *Xba*I*-Eco*RV gene cassette) from *G. max* (GenBank accession no. AY595413.1), FNS (as *Eco*RI-*Xba*I gene cassette) from *Petroselinum crispum* (GenBank accession no. AY230247.1) and F3′H (as *Eco*RV-*Bam*HI gene cassette) from *Arabidopsis thaliana* (GenBank accession no. Q9SD85). Compatible restriction sites were added at each gene cassette end, in order to facilitate construction of the recombinant flavonoids gene clusters, as well as ribosome binding sites at the 5′-ends.

All constructed plasmids (**Figure 1**) described below were verified by restriction enzymes digestions and also by sequencing of the cloned regions. *S. albus* producing clones were confirmed by PCR by the use of oligonucleotides designed to amplify the junction site at the first two common genes (TAL and 4CL): 5′-GTGATCGAGCTGGACATGAA-3′ as the forward primer and 5′-GGCGTCCACGAGGTGC-3′ as the reverse primer.

### Construction of pAPI

The plasmid pAPI is a pIAGO derivative (**Table 1**) containing the P_ermE^∗^_ and the five genes responsible for apigenin *de novo* biosynthesis. All synthetic gene cassettes were independently cloned in pUC57 and those plasmids were named pLMF1 (pUC57 containing TAL gene), pLMF2 (4CL), pLMF3 (CHS), pLMF5 (CHI), and pLMF-FNS (FNS). Additionally, TAL gene was subcloned into vector pSL1180 as *Hind*III-*Bam*HI (pLMF7) to start with the cloning strategy. 4CL gene (from pLMF2) was cloned into pLMF7 as *Pst*I-*Bam*HI gene cassette, generating pLMF8. Next step was subcloning FNS gene cassette from pLMF-FNS into pLMF3 as an *Eco*RI-*Xba*I DNA fragment, giving rise to pNGM1. The two gene cassettes from pNGM1 (CHS and FNS) were subcloned together into pLMF8 as *Sac*I-*Bam*HI DNA band, in order to get the first four genes together in a plasmid (pNGM2). Finally, CHI gene was subcloned into pNGM2 as *Xba*I-*Bam*HI DNA fragment resulting in the generation of pNGM3, which contains the five genes required for apigenin biosynthesis.

As the expression host was *Streptomyces*, a further subcloning was required, and the *Bgl*II-*Bam*HI DNA fragment from pNGM3 carrying these five genes was finally subcloned into a derivative of the bifunctional replicative vector pIAGO, under the control of P_ermE^∗^_, giving rise to the final plasmid pAPI.

### Construction of pLUT

The plasmid pLUT (**Table 1**) directs the biosynthesis of luteolin and contains the six required genes (TAL, 4CL, CHS, CHI, FNS, and F3′H), as well as the P_ermE^∗^_. This plasmid requires only one more gene (F3′H) than those ones present in pAPI. Therefore, pNGM3 was used for the construction of pLUT. The gene cassette F3′H was subcloned into pNGM3 as an *Eco*RV-*Bam*HI DNA fragment. This plasmid was named pNGM5 and contains all six genes. These genes were further subcloned as a *Bgl*II-*Bam*HI DNA fragment into the pIAGO vector, in order to achieve expression in *S. albus*, giving rise to plasmid pLUT.

### Construction of pERI

The plasmid pERI (**Table 1**) contains the P_ermE^∗^_ and the five genes required for the biosynthesis of eriodictyol. These genes are the ones encoding for TAL, 4CL, CHS, CHI, and F3′H. As all the genes but the one coding for FNS are the same ones required to produce luteolin, the construction of the plasmid directing the biosynthesis of eriodictyol was based on the previously described plasmid, pNGM5. This plasmid was digested with *Eco*RI-*Xba*I, and then blunt-ended with Klenow fragment, to eliminate the gene cassette FNS. This new pSL1180 derivative plasmid was named pNGM4. In order to express these five genes in *S. albus*, they were subcloned in the replicative vector pIAGO as a *Bgl*II-*Bam*HI DNA fragment, giving rise to the final plasmid, pERI.

### Extraction and Analysis of Flavonoids

Spores from the different *S. albus* J1074 recombinant clones harboring pAPI, pERI, pLUT, and pIAGO (negative control) were incubated during 184 h in 25 ml of liquid production medium R5A. Flavonoids extraction was carried out using three volumes of ethyl acetate and extracting separately the supernatant and the disrupted mycelium pellet (after acetone treatment). These extraction mixtures were incubated for 1 h in orbital shaker at room temperature. After this incubation, the organic phases were filtered, mixed together and concentrated by rotary evaporation and kept at -20°C for later use. All cultivation experiments were carried out three times for each strain or analysis.

Dry extracts were dissolved in 200 μl of methanol:DMSO (1:1), filtrated (0.4 μm cellulose filters) and analyzed by liquid chromatography-electrospray ionization mass spectrometry (HPLC-ESI-MS/MS, Agilent technologies 1290 Infinity, Triple Quadrupole). This chromatography was carried out using a Zorbax Eclipse Plus C18 column (50 mm × 2.1 mm, 1.8 μm) in the positive ion mode. The analytes were eluted at a flow rate of 0.3 mL/min using a gradient of 0.1% (v/v) formic acid in water (A) and 0.1% (v/v) formic acid in acetonitrile (B) at 0–10% of B for 1 min, which was increased to 35% for 3 min and maintained at 35% for 1 min; then increased to 80% for 3 min and maintained at 80% for 2 min and finally decreased to 10% for 1 min.

Flavonoids quantification was carried out in multiple reaction monitoring (MRM) mode in MS/MS. To accomplish this, the following ion sets were selected to detect the transitions of the parent ions to the product ions specific to the analytes: naringenin 272 > 119 Da and 272 > 151 Da; apigenin 270 > 117 Da and 270 > 150 Da; eriodictyol 288 > 135 Da and 288 > 150 Da; luteolin 286 > 131 Da and 286 > 151 Da. Pure flavonoid standards were purchased from Sigma Aldrich (apigenin, eriodictyol, naringenin) and VWR (luteolin).

### Spore Conditioning Experiments

Spore conditioning was carried out by incubating during 10 days a suspension of 10^8^ or 10^9^ spores/mL in 5 mL R5A medium, at 30°C and 250 rpm, in 50 mL Falcon tubes (final spore concentrations of 10^5^ and 10^6^ spore equivalent/mL respectively). This incubation was carried out in order to force spore germination in a high density culture, which induces programed cell death in mycelium I (MI), releasing the developmental signals that induce the differentiation (conditioning) of the MI cellular segments that remained viable into a new mycelium (MII), that is the secondary metabolite producer (Manteca et al., 2008). Due to the high density, this conditioned mycelium (MII), cannot growth too much, and remains quiescent until it is inoculated into a fresh medium (Manteca et al., 2008).

Thirty μL of the conditioned cultures (equivalent to 3 × 10^6^ or 3 × 10^7^ spores) were inoculated in 25 mL R5A medium in a 250 mL buffled flask (triplicates). As a control, simultaneously, other flask triplicates with 25 mL R5A medium were inoculated with our standard conditions, 30 μL of a spore stock solution (at 10^10^ spores/mL), which represents a final spore concentration in the flasks of 10^7^ spores/mL All these nine flasks were incubated at 30°C and 250 rpm during 168 h, and samples were taken every 24 h: 20 μL samples for confocal microscopy; 180 μL samples for protein quantification using the Bradford method (Bradford, 1976) after boiling in 0.5 M NaOH for 10 min and after removing cellular debris by centrifugation (at 7740 × *g* for 15 min); and 1.5 mL for apigenin extraction and HPLC–MS quantification.

### Feeding Experiments

Two hundred and fifty ml Erlenmeyer flasks with 25 mL R5A medium were inoculated with 10^7^ spores/mL of *S. albus*-pAPI and incubated at 30°C and 250 rpm during 48 h, according with previous literature (Park et al., 2009). At this time point, amounts of different precursors were added to achieve final concentrations of 1.2 mM *p*-coumaric acid, 13.5 mM sodium malonate, 1.2 mM *p*-coumaric acid plus 13.5 mM sodium malonate, or 0.1 mM naringenin in the corresponding flasks (triplicates). 1.5 mL of each culture was extracted at 96 h, as described in Section “Extraction and Analysis of Flavonoids,” and apigenin was quantified by HPLC–MS. These final precursors concentrations were selected based in literature (Park et al., 2009, 2010).

### Laser Scanning Fluorescence Microscopy

*Streptomyces albus* cultures from 24 h until 96 h were stained using the LIVE/DEAD BacLight Bacterial Viability Kit (Invitrogen), and observed under a Leica TCS-SP2-AOBS laser scanning microscope at wavelengths of 488 and 568 nm for excitation and 530 and 630 nm emission (optical sections of about 0.2 μm). Images were mixed with the Leica Confocal Software. In these conditions, living mycelium appears green colored (SYTO9 staining), while dying mycelium appears red (PI staining). Septa are visible as discontinuities in the SYTO9/PI stained hyphae; MI is fully compartmentalized, while MII (the secondary metabolite producer mycelium) is multinucleated with sporadic septa (Manteca et al., 2008).

## Results

### Heterologous Production of Apigenin

The flavone apigenin is generated due to the action of the enzyme FNS (flavone synthase) on the important flavonoid precursor naringenina (**Figure 1**). In order to produce the plant metabolite naringenin in microorganisms, four enzymes are required: TAL, 4CL, CHS, and CHI (**Figure 1**). Four synthetic genes coding for these enzymes (adapted to the transcription and translation characteristics in prokaryotes as *S. albus*), together with a synthetic gene coding for FNS, were cloned in a replicative high-copy number shuttle vector for *E. coli–Streptomyces*, under the control of P_ermE^∗^_ (see Materials and Methods). The final plasmid construction, pAPI, was transformed and successfully expressed in the actinomycete *S. albus*, as a method to achieve future industrial production of this important flavonoid.

**FIGURE 2:**
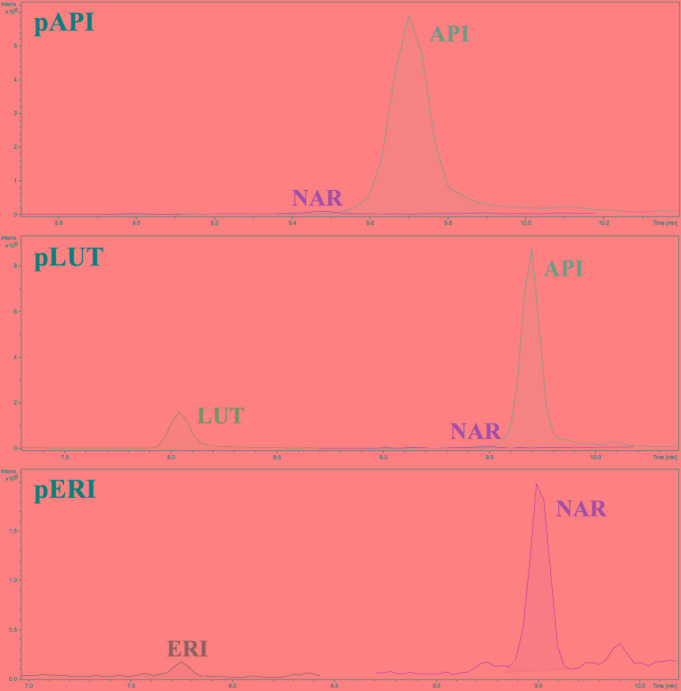
**HPLC–MS chromatograms showing the m/z peaks corresponding to naringenin (NAR), apigenin (API), luteolin (LUT), and eriodictyol (ERI).** In the corresponding *S. albus* strains.

Cultures of *S. albus*-pAPI in R5A liquid medium were analyzed by HPLC–MS chromatography in MRM in MS/MS mode, in order to identify and quantify the final product, apigenin, as well as its intermediate precursors naringenin and *p*-coumaric acid (**Figure 1**). *p*-Coumaric acid is the second common metabolite in flavonoids biosynthesis, after transformation of the initial aromatic amino acid precursor in plants (L-Phe) or in microorganisms (L-Tyr) (**Figure 1**). The three compounds (*p*-coumaric acid, naringenin, and apigenin) were detected in these analyses and their yields were quantified using commercial pure standards (see Materials and Methods). Production rates for apigenin synthetized by *S. albus*-pAPI were 0.08 mg/L. Its precursor naringenin was produced at lower level (0.014 mg/L), and the *p*-coumaric acid initial precursor for this pathway was observed at higher concentration, reaching levels of 0.774 mg/L (**Figure 2**). Negative control strain harboring the empty vector, *S. albus*-pIAGO showed no flavonoids in their HPLC–MS analyses.

**FIGURE 3:**
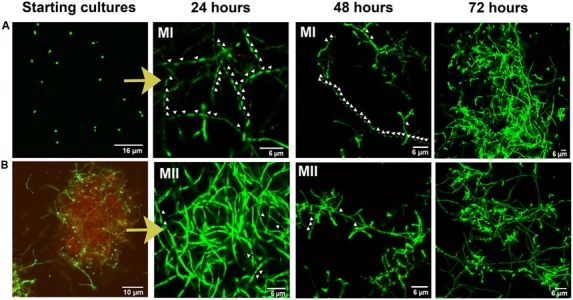
**Confocal laser-scanning fluorescence microscopy analysis (SYTO9/PI staining) of *Streptomyces albus*-pAPI development in liquid cultures under two different conditions. (A)** morphology of control cultures inoculated with a spore preinoculum. **(B)** Morphology of conditioned cultures inoculated with a high-density pre-grown culture in which programed cell death processes was induced. Arrows indicate mycelium septa. MI: type I mycelium (compartmentalized mycelium); MII: type II mycelium (multinucleated mycelium, secondary metabolite producer).

**FIGURE 4:**
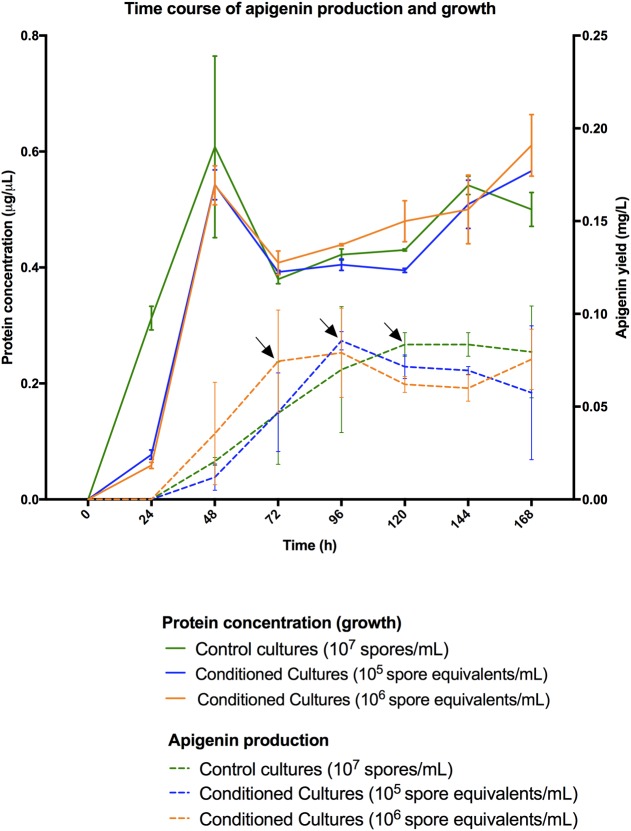
***Streptomyces albus*-pAPI growth curves (solid lines) and apigenin production curves (dashed lines) for three different types of cultivation experiments (mean values from three independent triplicate flasks).** Green colors correspond to control cultures with 10^7^ spores/mL. Blue colors correspond to conditioned cultures with a preinoculum of 10^5^ spore equivalents/mL. Orange colors correspond to conditioned cultures with a preinoculum of 10^6^ spore equivalents/mL. Apigenin production is anticipated in conditioned cultures, even with a delayed growth during the first 24-h, and achieving same production levels 1 and 2 days in advance, respectively. Maximum production levels for apigenin titers are labeled by arrows.

Differentiation of a multinucleated mycelium (MII) conditions secondary metabolism production (Manteca et al., 2008). Consequently, we tested the possibility to further improve apigenin production levels, or even anticipate time of maximum production, as a way to save money in future industrial fermentations, based in the improvement of the MII differentiation. Spore conditioning experiments were carried out with *S. albus*-pAPI (see Materials and Methods). Briefly, germination in dense spore inocula force programed cell death in the vegetative MI, releasing the developmental signals that induce the differentiation (conditioning) of the MI cellular segments that remained viable into a new mycelium (MII, secondary metabolite producer). Due to the high density, this conditioned mycelium (MII) remains quiescent until it is inoculated into a fresh medium (Manteca et al., 2008). Conditioning times longer that these 10 days did not show extra advantages with respect to final effect in generation of quiescent MII. Also, after these 10 days, the obtained mycelium II remains quiescent and conditioned during a long time (at least months), ready to be used in different production batches (Manteca et al., 2008). Development and apigenin production were compared in cultures inoculated with conditioned and non-condtioned spores (**Figures 3, 4**). These experiments showed that inocula from conditioned cultures contained high amounts of dead mycelium pellets (red staining), non-germinated spores, and quiescent MII (green staining) (**Figure 3B**). By contrast, control cultures (non-conditioned inocula) contained only spores (**Figure 3A**). After 24 h incubation, control flasks showed normal development toward MI (**Figure 3A**), whereas conditioned flasks at this point showed a high proportion of producing MII (**Figure 3B**). Control flasks did not show a high proportion of MII until 72 h (**Figure 3A**). Accordingly, apigenin production was faster and the maximum production levels earlier in conditioned flasks (72 h for 10^6^ conditioned spore equivalent/mL flasks and 96 h for 10^5^ conditioned spore equivalent/mL flasks) than in the control flasks (120 h, 10^7^ spores/mL) (**Figure 4**).

**FIGURE 5:**
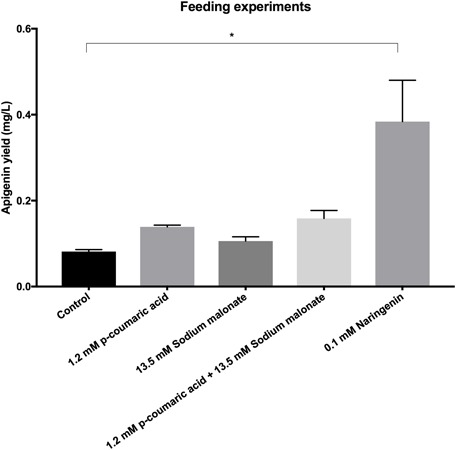
**Apigenin production titers in cultures of *S. albus*-pAPI without feeding (control), and with 1.2 mM *p*-coumaric acid, 13.5 mM sodium malonate, 1.2 mM *p*-coumaric acid plus 13.5 mM sodium malonate, or 0.1 mM naringenin final concentrations.** Only feeding with naringenin caused a significant 468% increase in apigenin biosynthesis with respect to control conditions. ^∗^Statistically significant differences (*p* < 0.1) by one-way ANOVA test.

Finally, in order to try to increase apigenin final production titers, feeding experiments were carried out (**Figure 5**). Feeding with coumaric acid, sodium malonate or both together did not increased significantly final apigenin titers (0.139, 0.106, and 0.159 mg/L apigenin; in contrast to 0.082 mg/L in control without feeding). However, feeding with 0.1 mM naringenin caused a statistically significant increase in apigenin production levels, achieving 0.384 mg/L.

### Heterologous Production of Eriodictyol

The flavanone eriodictyol is a hydroxylated form of the flavanone precursor naringenin. For its biosynthesis, only one extra enzymatic activity is required, the F3′H (flavonoid 3′-hydroxylase) at ring B (**Figure 1**), instead of the FNS oxidoreductase. Final replacement in pAPI plasmid of the gene cassette coding for FNS by the new one coding for F3′H generated a vector containing the five genes (TAL, 4CL, CHS, CHI, and F3′H) required for eriodictyol biosynthesis in *S. albus* under the control of P_ermE^∗^_ (see Materials and Methods).

This new recombinant plasmid, pERI, was transformed in *S. albus* protoplasts, and cultures from positive recombinant strains (in R5A liquid medium) were analyzed by MRM chromatography. These experiments demonstrated that recombinant plasmid pERI was able to redirect the biosynthesis from apigenin to eriodictyol in *S. albus*, using as common precursor the naringenin already detected in previous experiments. However, production levels for eriodictyol in these extracts were very low under these conditions (0.002 mg/L). However, higher levels of the naringenin precursor (0.037 mg/L) were observed. The initial precursor *p*-coumaric acid was also analyzed, showing production levels of 1.256 mg/L (**Figure 2**).

### Heterologous Production of Luteolin

Luteolin biosynthesis in microorganisms requires the activity of six enzymes: TAL, 4CL, CHS, CHI, FNS, and F3′H. The genes coding for these enzymes were cloned in the same replicative high-copy number shuttle vector for *E. coli–Streptomyces* (see Materials and Methods), under the control of the promoter P_ermE^∗^_, generating the final plasmid pLUT. This plasmid was transformed into *S. albus* protoplasts.

Clones of this recombinant strain were cultivated in R5A liquid medium and flavonoids productivity was quantified using HPLC–MS in their extracts. Luteolin was detected with production rates of 0.09 mg/L) but its precursor apigenin was observed at higher levels (0.085 mg/L) (**Figure 2**). As in previous experiments, a higher accumulation of the initial precursor *p*-coumaric acid was detected (0.872 mg/L).

## Discussion

In this work, *S. albus* has been able to *de novo* biosynthetize four flavonoids: naringenin (a flavanone precursor in this family of nutraceuticals), apigenin (its oxidized flavone derivative), luteolin (a 3′-hydroxylated apigenin) and eriodictyol (a 3′-hydroxylated version of the flavanone naringenin).

*De novo* naringenin production has been previously described in recombinant strains of *E. coli* (Hwang et al., 2003; Miyahisa et al., 2006; Santos et al., 2011)*, S. cerevisiae* (Yan et al., 2005; Trantas et al., 2009), and *S. clavuligerus* (Álvarez-Álvarez et al., 2015). Other authors also achieved its production in other microorganisms, but after feeding with precursors (Park et al., 2009).

Apigenin was previously produced *de novo* only in *E. coli* (Miyahisa et al., 2006). Here, the complete biosynthetic pathway was cloned in *E. coli* and production levels reached up to 13 mg/L. However, the addition to culture medium of the main precursor L-tyrosine at 543 mg/L (3 mM) was required (Miyahisa et al., 2006) (**Table 3**). In a more recent work, apigenin yields were increased to 23 mg/L in *E. coli*. But, as in the work developed by Miyahisa et al. (2006), its synthesis needed the supplementation with *p*-coumaric acid (Lee et al., 2015). Higher production levels in *E. coli*, 110 mg/L, have been described after feeding with *p*-coumaric acid and malonate (Leonard et al., 2008). This flavone was also produced in *S. venezuelae* at 1.5 mg/L, but only after feeding with 0.5 mM (136 mg/L) naringenin a *S. venezuelae* strain which contained only the gene coding for FNS oxidoreductase (Park et al., 2010) (**Table 3**).

**Table 3:** List of heterologous microbial hosts for biosynthesis of flavanones and flavones in the literature and estimated production titers.

Heterologous flavonoid produced	Host	Externally fed precursor	Production titers (mg/L)	Reference
Apigenin	*E. coli*	L-Tyrosine	13	Miyahisa et al., 2006
	*E. coli*	*p*-Coumaric acid	23	Lee et al., 2015
	*S. venezuelae*	Naringenin	1.5	Park et al., 2010
	*S. venezuelae*	*p*-Coumaric acid	15.3	Park et al., 2011
	*S. cerevisiae*	*p*-Coumaric acid	3.5	Leonard et al., 2005
	*E. coli*	*p*-Coumaric acid and malonate	110	Leonard et al., 2008
Eriodictyol	*E. coli*	Caffeic acid	0.03	Leonard et al., 2006
	*E. coli*	L-Tyrosine	107	Zhu S. et al., 2014
	*S. cerevisiae*	Caffeic acid	6.5	Yan et al., 2005
	*S. cerevisiae*	Caffeic acid	20	Leonard et al., 2005
	*E. coli*	*p*-Coumaric acid and malonate	50	Leonard et al., 2008
Luteolin	*S. cerevisiae*	Caffeic acid	2	Leonard et al., 2005
	*E. coli*	*p*-Coumaric acid and malonate	4	Leonard et al., 2008


In this work, however, the complete biosynthetic pathway for apigenin was heterologous expressed in *S. albus*. Besides, it was not supplemented with any precursor, although the levels of apigenin obtained (0.089 mg/L) were smaller than in other previously described works. Only small amounts (0.001 mg/L) of its immediate precursor naringenin were detected, indicating that the activity of the plant oxidoreductase enzyme FNS is good in this microorganism.

In the case of eriodictyol, according to the literature, its biosynthesis in actinomycetes had not been described before. However, two research groups have reported production of this flavanone in *E. coli* from glucose or after feeding with *p*-coumaric acid plus malonate, caffeic acid or L-tyrosine, reaching production levels of 50, 5.70, 0.02, and 42.6 mg/L respectively (Leonard et al., 2007, 2008; Zhu S. et al., 2014) (**Table 3**). In both cases, metabolic engineering in *E. coli* was carried out afterward to enhance the availability of the flavonoids precursor malonyl-CoA, increasing the eriodictyol production levels to 52 and 107 mg/L respectively (Leonard et al., 2007; Zhu S. et al., 2014) (**Table 3**). In our case, despite the low eriodictyol production levels in *S. albus*, this biosynthesis is *de novo* and has been achieved in an actinomycete for the first time. This low production levels, as well as the accumulation of its precursors naringenin and *p*-coumaric acid, show a low activity for the hydroxylase F3′H (see below).

In the case of the flavone luteolin, its heterologous biosynthesis has been described in *E. coli* (4 mg/L) and *S. cerevisiae* (2 mg/L) after feeding with *p*-coumaric acid plus malonate, or caffeic acid respectively (Leonard et al., 2005, 2008) (**Table 3**). In our case, we demonstrate its *de novo* biosynthesis in *Streptomyces*. Nonetheless, as in the case of eriodictyol, luteolin yields were low. This was expected after the observed eriodictyol results, as luteolin biosynthesis requires also the activity of F3′H hydroxylase (**Figure 1**).

These experiments showed that F3′H enzyme in *S. albus* is not able to completely transform naringenin into eriodictyol nor apigenin into luteolin. In *S. albus*-pLUT, no eriodictyol was observed, indicating that the FNS enzyme is quite more efficient than F3′H, as apigenin is detected in higher amounts here than eriodictyol. This idea is supported also by the fact that eriodictyol levels in *S. albus*-pERI were almost 300 times lower than apigenin levels in *S. albus*-pAPI. Other authors have described also that P450 hydroxylases like F3′H from eukaryotic origin may present low solubility in prokaryotic hosts, as in this case *S. albus*, because a membrane anchorage domain may not be suitable in prokaryote systems (Park et al., 2011). The hydroxylase F3′H is a cytochrome P450-dependent monooxygenase which requires its integration into the membrane of the endoplasmic reticulum of plant cells as well as the presence of a P450-reductase carrying electrons from a donor to a NADPH P450 heme-core complex. Functional expression of these plant membrane-bound enzymes in microorganisms is a challenge due to several factors, such as protein insolubility (prokaryotes do not have endoplasmic reticulum membranes to bound) and cofactor incorporation (Burbulis and Winkel-Shirley, 1999; Chang et al., 2007; Fowler and Koffas, 2009). It has been shown that microbial expression of this family of enzymes is enhanced by engineering P450 reductases, in a way that these reductases are fused to the corresponding P450-dependent monooxygenase, and the membrane binding domain of these ones is deleted to increase solubility in prokaryotic cytoplasm. Following this strategy, soluble and functional chimeras have been obtained (Leonard et al., 2006; Leonard and Koffas, 2007; Zhu S. et al., 2014). These reasons for the monooxygenases counterparts could explain the very low F3′H activity shown in this study as shown with the low lutein and eriodictyol levels in *S. albus*. Therefore, these studies in *S. albus* will pave the way in order to conduct further enzyme engineering in this type of enzymes, to achieve higher production titers (Zhu S. et al., 2014) (**Table 3**).

Finally, we have seen accumulation of *p*-coumaric acid in our three producing strains, the second metabolite in the pathway (after TAL activity on L-Tyr), which is common to all flavonoid biosynthetic pathways. *p*-Coumaric acid is converted to coumaroyl-CoA by the action of 4CL enzyme, which requires malonyl CoA to synthetize the corresponding flavonoid (naringenin chalcone in our case, **Figure 1**). Malonyl-CoA is therefore a limiting factor in flavonoid production, as it is also precursor for fatty acids biosynthesis (Winkel-Shirley, 2001). In fact, different authors consider that this influx of malonyl-CoA is the biggest bottleneck in the flavonoids biosynthesis, since three molecules of malonyl-CoA are needed to generate a molecule of eriodityol, apigenin, or luteolin in our case. Basal levels of this metabolite inside cells are relatively low, as it is regularly consumed during fatty acids biosynthesis, so low intracellular malonyl-CoA availability may restrict the efficient production of flavonoids in both *E. coli* and *Streptomyces* strains (Santos et al., 2011; Rathnasingh et al., 2012). Accordingly, diverse described strategies to increase flavonoids yields focused on increasing the intracellular pool of malonyl-CoA by overexpressing the acetyl-CoA carboxylase enzyme complex (ACC) in the host strain (Hwang et al., 2003; Miyahisa et al., 2006; Park et al., 2011; Wang et al., 2011). ACC overexpression increased cell concentration malonyl-CoA in a 278% in relation to the *E. coli* wild strain (Zhu S. et al., 2014). In our case, with so high intracellular *p*-coumaric acid accumulation in comparison to final flavonoids, we can hypothesize that the reason may be also a low supply of malonyl-CoA, due to its consumption by fatty acids biosynthesis in *S. albus.* However, feeding with pure 13.5 mM sodium malonate final concentration in flasks did not generated a significant increase in production levels. The cause of this may be that initially identified similar genes in *S. albus* chromosome to *matB* (membrane malonate carrier protein) and *matC* (malonyl-CoA synthetase) from *S. coelicolor* counterparts (*sco2443, sco2445*) (Park et al., 2011), actually do carry out other enzymatic functions in *S. albus*, and therefore this bacterium cannot incorporate extracellular sodium malonate toward flavonoids biosynthesis. As this step is the bottleneck in flavonoids biosynthesis, addition of *p*-coumaric acid nor both precursors (*p*-coumaric acid plus sodium malonate) does not increase apigenin final titers (**Figure 5**). In order to check if feeding with the precursor naringenin was an alternative for increasing final apigenin levels, 0.1 mM of this flavanone were added to *S. albus*-pAPI cultures, and in this case, apigenin production levels were increased 468% (**Figure 5**). Once we have achieved biosynthesis of these four flavonoids in *S. albus*, future metabolic engineering in this actinomycete, where the genome information is available, will be able to increase carbon flux toward malonyl-CoA, most probably leading to higher flavonoids production levels. Also, it will be possible to enhance intracellular production levels for *p*-coumaric acid, or limit at some degree possible catabolism pathways or enzymes affecting these precursors or flavonoid intermediates.

With respect to other heterologous hosts for production of bioactives as flavonoids, actinomycetes as *S. albus*, do possess the same degree of available genetic tools for genome and metabolic engineering, in comparison to *E. coli*, but more suitable and easier genetic tools than in the case of the eukaryote *S. cerevisiae*, which has the disadvantage that two copies for each gene function must be modify. As an extra advantage, the huge genetic and metabolic richness derived from large actinomycetes chromosomes allows these bacteria to possess a high diversity of anabolic and catabolic pathways in charge of providing different precursors needed for heterologous biosynthesis of a wide fan of bioactives, as it has been described in “Introduction” (Newman and Cragg, 2007).

With respect to the Gram-negative *E. coli* host, production of nutraceuticals like flavonoids for the pharmaceutical industries is favored in Gram-positives as *S. albus.* The reason for this is that *S. albus*, as a canonical Gram-positive bacterium, lacks the presence of important endotoxins for human cells, as those derived from *E. coli* outer membrane. Industrial removal of these lipopolysaccharide toxins involves costly procedures (Lopes et al., 2010).

As an actinomycete, *S. albus* shows has a complex cell cycle that can be modified in order to further increase production yields of secondary metabolism heterologous bioactives. Secondary metabolism is activated during the MII stage (Manteca et al., 2008). In this work, we have carried out *de novo* production of apigenin in *S. albus*-pAPI strain by using three alternative cultivation approaches, with and without spore conditioning (Manteca et al., 2008). It is noticeable that similar production levels were obtained in conditioned and not conditioned cultures, but production was anticipated up to 2 days in the conditioned cultures, even with a delayed growth during the first 24-h. Consequently, conditioned cultures can be useful to manipulate production rates in eventual *S. albus* industrial fermentations, as it was reported in other streptomycetes (Sánchez and Braña, 1996; Rioseras et al., 2014).

## Author Contributions

LM and IG-d-R were involved in creation of the different recombinant plasmids and heterologous production strains. CV and FL were supervisors of these experiments. PY and AM contributed to the perform conditioning experiments. FL is principal investigator of the research project were these experiments were planned.

## Conflict of Interest Statement

The authors declare that the research was conducted in the absence of any commercial or financial relationships that could be construed as a potential conflict of interest.
